# Extracellular Electrophysiology in the Prostate Cancer Cell Model PC-3

**DOI:** 10.3390/s19010139

**Published:** 2019-01-03

**Authors:** Miguel Cabello, Haobo Ge, Carmen Aracil, Despina Moschou, Pedro Estrela, Jose Manuel Quero, Sofia I. Pascu, Paulo R. F. Rocha

**Affiliations:** 1 Department of Electronic Engineering, Escuela Superior de Ingenieros, University of Seville, 41004 Seville, Spain; mcabellov@gte.esi.us.es (M.C.); caracil@gte.esi.us.es (C.A.); quero@us.es (J.M.Q.); 2 Department of Chemistry, University of Bath, Claverton Down, Bath BA2 7AY, UK; H.Ge@bath.ac.uk (H.G.); S.Pascu@bath.ac.uk (S.I.P.); 3 Centre for Biosensors, Bioelectronics and Biodevices (C3Bio), Department of Electronic and Electrical Engineering, University of Bath, Claverton Down, Bath BA2 7AY, UK; D.Moschou@bath.ac.uk (D.M.); P.Estrela@bath.ac.uk (P.E.)

**Keywords:** prostate cancer signalling, PC-3 cells, electrical activity, calcium channel inhibitor

## Abstract

Although prostate cancer is one of the most common cancers in the male population, its basic biological function at a cellular level remains to be fully understood. This lack of in depth understanding of its physiology significantly hinders the development of new, targeted and more effective treatment strategies. Whilst electrophysiological studies can provide in depth analysis, the possibility of recording electrical activity in large populations of non-neuronal cells remains a significant challenge, even harder to address in the picoAmpere-range, which is typical of cellular level electrical activities. In this paper, we present the measurement and characterization of electrical activity of populations of prostate cancer cells PC-3, demonstrating for the first time a meaningful electrical pattern. The low noise system used comprises a multi-electrode array (MEA) with circular gold electrodes on silicon oxide substrates. The extracellular capacitive currents present two standard patterns: an asynchronous sporadic pattern and a synchronous quasi-periodic biphasic spike pattern. An amplitude of ±150 pA, a width between 50–300 ms and an inter-spike interval around 0.5 Hz characterize the quasi-periodic spikes. Our experiments using treatment of cells with Gd^3^⁺, known as an inhibitor for the Ca^2^⁺ exchanges, suggest that the quasi-periodic signals originate from Ca^2^⁺ channels. After adding the Gd^3^⁺ to a population of living PC-3 cells, their electrical activity considerably decreased; once the culture was washed, thus eliminating the Gd^3^⁺ containing medium and addition of fresh cellular growth medium, the PC-3 cells recovered their normal electrical activity. Cellular viability plots have been carried out, demonstrating that the PC-3 cells remain viable after the use of Gd^3^⁺, on the timescale of this experiment. Hence, this experimental work suggests that Ca^2^⁺ is significantly affecting the electrophysiological communication pattern among PC-3 cell populations. Our measuring platform opens up new avenues for real time and highly sensitive investigations of prostate cancer signalling pathways.

## 1. Introduction

Prostate cancer is one of the most common malignancy cancers diagnosed in men [1], especially in Western countries [2,3]. In the UK alone, about 50,000 men per year are diagnosed with prostate cancer [4]. The disease can develop when cells in the prostate start to grow and spread in an uncontrolled way. Also, owing to its difficulty in diagnosis, there is an urgent need for early prevention and efficient treatment to stop it spreading. Nonetheless, there is still little scientific understanding on the electrophysiology of the prostate cancer growth and metastatic patterns, thus delaying the development of new, targeted drugs for confinement and treatment of the tumour [5].

Currently, a reliable and useful in-vitro model for prostate cancer is cell culture [6]. Along with LNCaP and DU-145, PC-3 cells are considered the gold standard of prostate cancer cell culture lines [7,8]. PC-3 cells are proven to be reliable for growth rate and behaviour as a xenograft [9], and maintain genotype and phenotype when injected into mice [10,11]. Finally, PC-3 cell lines are hormone insensitive and present no AR or PSA mRNA/protein. As a highly aneuploid line, it duplicates after approximately 33 h. This proliferation is known to lead to high levels of oxidative stress [12]. Concomitantly, the scientific community has routinely attributed the transduction of oxidative stress to the universal second messenger Ca^2+^. The pH sensitivity in comparison to non-malignant cells is noticeable and being utilized in the development of novel anticancer drugs [13]. The routine use of this methodology is hindered by the expensive Ca^2+^ dyes toxicity and inability to perform long term Ca^2+^ imaging. 

Fluctuations of the membrane potential play a central role in cells of the nervous system. They are caused by the flux of primarily Na^+^, K^+^, Cl^−^, and Ca^2+^ ions along the gradient, controlled by the function of ion channel proteins. Gradients in ion channels are closely related to brain illnesses [14] cardiac arrhythmias [15] and the development of cancer [16,17,18,19,20,21]. Previous imaging and single cell studies investigated K^+^, Na^+^ and Ca^2+^ channels in human prostate cancer basal activity and proliferation [22,23] Consensus on the role of Ca^2+^ channels during prostate cancer proliferation seems to exist as demonstrated by Zhang and colleagues through the use of Ca^2^⁺-permeable channel TRPM8 [24,25] and by others trough a permeable channel to Ca^2+^, TRPC6 [26] and other oxidative stresses [27].

Moving ions across the membrane of cells gives rise to minute electrical fluctuations, even in electrically quiescent cells such as Glia cells [28,29,30,31,32]. Hence, we hypothesize that electrical monitoring of prostate cancer cells would also be possible, not through using expensive and sometimes toxic methods such as fluorescence, but instead using a low cost, ultra-sensitive electrical recording setup to measure a whole population of non-neuronal cells.

To electrically detect a population of living cells, multi-electrode arrays (MEAs) are used as the technology of choice. A MEA consists of a group of electrodes on a substrate which allow a close contact with cells in culture medium. The first MEA used to monitor the electrical activity of cells culture was created around 1970 [33]. Since then, most of the efforts related to MEAs have been focused on improving the concentration of electrodes and their electrical characteristics [34,35,36,37,38]. A major goal of electrode fabrication for application in MEAs is to achieve a low impedance and high capacitance, as it results in a higher signal-to-noise ratio; low impedance becomes particularly challenging when the planar electrode dimensions are miniaturized down to the micrometre scale. Therefore, researchers are focusing on increasing the effective surface area by modifying the electrode with porous conducting materials such as Pt black, Au nanostructures and carbon nanotubes. By modifying the surface, the impedance of the electrode is reduced, leading to improved electrical recordings. In this respect, we have decreased the impedance by using extreme large electrode areas of mm^2^ and have shown a noise level as low as 0.3 µV_pp_ [31]. An extremely low-noise measuring system allowed us to detect minute, yet constantly occurring and functional, membrane capacitive current oscillations across large populations of cells, such as e.g., C6 glioma cells [32].

In this paper, we show that the reliable prostate cancer cell model PC-3 can be electrically monitored using the same extremely high signal-to-noise electrophysiological recording system. We recorded the electrical activity of populations of PC-3 cells over time and show that two typical and consistent behaviours occur. The first one relates to the basal activity and is manifested as asynchronous and sporadic electrical spikes. The second is a collaborative event and is manifested through the observation of a quasi-periodic electrical spike-pattern. By using specific inhibitors, we show that the routinely observed quasi-periodic spikes relate to Ca^2+^ ions cooperatively traveling through a population of thousands of cells. Our findings also suggest the applicability of our cells-on-a-chip system for reliable in-vitro testing of human-relevant prostate cancer models such as PC-3 cells for in-vivo applications.

## 2. Materials and Methods

### 2.1. General Cellular Culturing Method

PC-3 cells were cultured using standard methodologies, at 37 °C in a humidified atmosphere in air and harvested once a confluence of over 70% had been reached. PC-3 cells were cultured in Roswell Park Memorial Institute (RPMI) 1640 medium. The media contained 10% foetal calf serum (FCS), 0.5% penicillin/streptomycin (10,000 IU mL^−1^/10,000 mg mL^−1^) and 1% 200 mM L-glutamine. All culturing and imaging steps were performed in the absence of phenol red-based additives. The supernatant containing dead cell matter and excess protein were aspirated. The live adherent cells were then washed with 10 mL of phosphate buffer saline (PBS) solution twice to remove any remaining media containing FCS, which may inactivate trypsin. Cells were incubated in 3 mL of trypsin solution (0.25% trypsin) for 5 to 7 min at 37 °C. After trypsinisation, 6 mL of medium containing 10% serum was added to inactivate the trypsin and the solution was centrifuged for 5 min (1000 rpm, 25 °C) to remove any remaining dead cell matter. The supernatant liquid was aspirated and 5 mL of cell medium (10% FCS) was added to the cell matter left behind. Cells were counted using a haemocytometer and then seeded as appropriate, either on a microchip, or on a cellular plate suitable for optical imaging.

### 2.2. Standard Cellular Viability Assays in Water 

To assess the cellular viability over the duration of experiment, in a parallel setting, standard MTT assays (i.e., colorimetric assays for measuring cell metabolic activity) were performed. For this, PC-3 cells were plated (7 × 10^3^ cells per well) in a 96-well plate and left for 48 h to adhere fully. For a cellular viability estimation (denoted IC50 estimations by MTT assays), cells were incubated with aqueous gadolinium chloride and tested for 20 min at 37 °C. Concentrations used were 250, 100, 50, 10, 1, 0.5, 0.1 µM, 0.001 µM (1% water, 99% RPMI medium containing 10% FCS at standard concentration of the cell line). Subsequently, cells were washed three times with PBS and 100 µL of MTT was added (0.5 mg mL^−1^, 10% PBS:SFM), followed by a 2-h incubation. Following aspiration, 100 µL of DMSO was added and the 96 well plates were read at an ELISA plate reader (Fluostar Omega BMGLabTech, Aylesbury, UK). Data emerged from at least three consistent results and IC_50_ values were calculated using Origin 9 (Wellesley Hills, MA, USA). However, when the effect of GdCl^3^ was evaluated in aqueous conditions, the IC_50_ value at 20 min could not be determined because no significant cytotoxicity effect is induced by aqueous gadolinium chloride in 20-min incubation with given concentrations. 

### 2.3. MEA Experimental Details

The MEA chip used for the experiments consists of a 1 mm thick silicon/silicon dioxide substrate and round shaped Au electrodes of 50 nm thickness and 2 mm apart. Au was evaporated on top of a 8 nm Cr adhesion layer through a shadow mask. The electrode area is 1 mm^2^. Then, a previously drilled piece of PMMA was glued to the substrate with the electrodes, which is used as a well to contain the solution. The gold electrodes, which have an area of 1 mm^2^, are located inside the well and connected with a small strip-line of 0.2 mm to the contact pad outside the well. The area of the strip-line can be disregarded with respect to the area of the electrodes. The Au plated contact pins outside the well were purchased from Distrelec (Bremen, Germany), rated at 3 A with a length of 24.64 mm and type SPA-2D, allowing the connection to the chip with the measurement equipment. After autoclaving the whole system, cells are deposited with the medium over the electrode. Recordings start at least 3 h after cell deposition. A total of 0.1 million cells were prepared for its culture in the described MEA. The whole system was autoclaved before each experiment. A total of 280 µL (0.1 M) were loaded onto the MEA chip. Devices were placed in the incubator and monitored continuously up to 3 days. The whole experiment was repeated three times with the same conditions. The well was loosely covered with a lid to prevent evaporation of the medium. After filling, the system was put into an incubator (Midi 40, Thermo Scientific, Schwerte, Germany). This system assures the presence of enough cell culture medium to keep the cells viable over more than 24 h without medium change.

The current between two Au electrodes was measured using a low-noise current amplifier (SR570, Stanford Research, Sunnyvale, CA, USA) and a dynamic signal analyser (35670A, Agilent, Frankfurt, Germany). The data from the signal analyser was extracted with a homemade LabVIEW acquisition software using a high speed USB/GPIB interface converter from Keysight Technologies (Frankfurt, Germany) and processed using Matlab R2017b. To minimize drift, the current amplifier was calibrated and the setup was stabilized for at least 2 h before measuring. The current was recorded as a function of time by using zero bias on the electrodes. The use of a Faraday cage and low noise wires assure a minimized external interference. 

Optical micrographs taken with an epifluorescence microscope (Nikon, Surrey, UK) showed that the PC-3 cells adhered and covered the whole electrode and substrate. Because of the large electrode area, the recorded current was not from a single cell but from the superposition of signals of all cells coupled to the electrode. Hence, we recorded the activity of a PC-3 cell population, and findings are discussed below.

## 3. Results and Discussion

### 3.1. Sensor, Cell Adhesion and Viability

**Figure 1:**
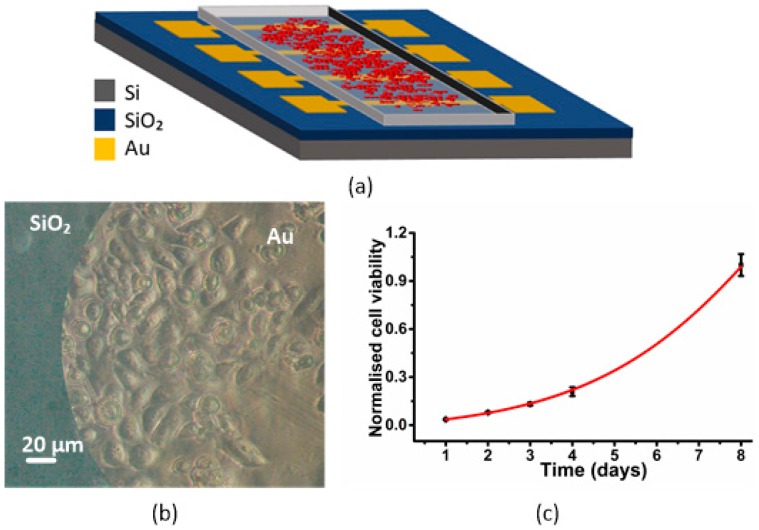
(**a**) Drawing of the transducer used for the culturing of PC-3 cells. PC-3 cells are represented as red circles within the culture medium. (**b**) PC-3 cells adhered to the gold electrodes 3 days after being deposited. (**c**) Cell viability describing a typical exponential PC-3 cell growth in the MEA device.

Figure 1a illustrates the MEA chip used during the experiments, in which red circles represent the cells inside the chip. The chip is composed of four pairs of circular electrodes, which are optimized to record the electrical signals of cells populations adhered to them [39,40]. Pairs of electrodes comprise one measuring electrode and one counter electrode. To better understand the recording system, we have modelled the equivalent circuit based on the charge transfer resistance, R_D_, in parallel with the Helmholtz-Gouy-Chapman double layer capacitance, C_D_, which is in series with the spreading resistance, R_C_. The counter electrode has a similar circuit. The path between the sensing and the counter electrode is large, making the impedance, Z_seal_, very high. Cells generate a voltage, v_s_(t). Our approach measures the current i_s_(t) using a transimpedance amplifier. The output voltage is then given by v_o_(t):

(1) $$vo\left(t\right)=\;-R_{F}\;i_{s}\left(t\right)$$

where R_F_ is the feedback resistance. The model used in these experiments has been explained by Medeiros et al. [39]. The detected current is given as:

(2) $$i_{s}\left(t\right)=\;\frac{dv_{s}}{\mathrm{dt}}\cdot C_{D}\left(1-e^{-\frac{t}{\tau }}\right),\;\mathrm{with}\;\tau =R_{c}R_{D}$$

In this case, τ is the time constant for the charging or discharging of the network. C_D_ acts as a multiplying factor, which affects the spatial resolution, but also allows the amplification of the signal thanks to the rescaling of C_D_ due to the use of large-area electrodes. Changes on the extracellular potentials affect the current, which is the derivative of the acquired voltage signal [41]. 

Figure 1b shows the adhesion of cells to the substrate and the gold electrodes. Figure 1c shows cell viability in the developed sensors. For cell viability experiments, 1 × 10⁵ cells were added and incubated for 24 h. The growth of cells was monitored every day. The number of cells on eight different electrodes were counted and averaged. The results show the normalized cell numbers in 1, 2, 3, 4 and 8 days. It shows no toxicity in our recording system and that PC-3 cells can attach and proliferate in normal conditions. Each spot was calculated from the average of the eight different electrodes on the chips. The error reported was the standard error of the mean and is shown as ±S.E.M (see Table 1).

### 3.2. Electrical Activity of PC-3 Cells

**Figure 2:**
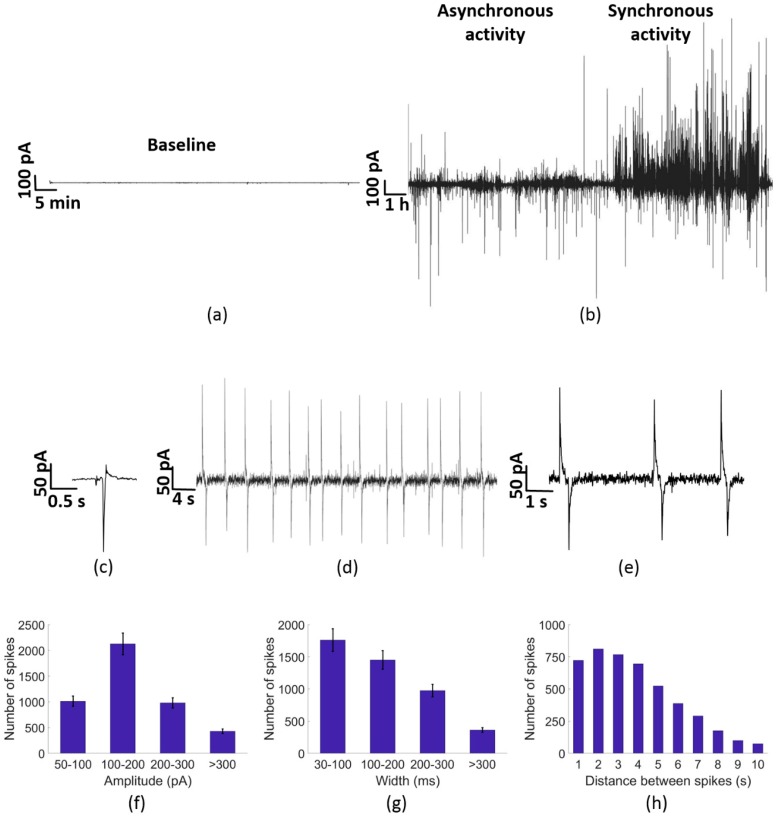
Electrical activity of a PC-3 cells culture on a Si/SiO_2_/Au substrate chip. (**a**) Baseline measured on the chip with only cell medium. (**b**) Most representative electrical activity of PC-3 cells during the experiment, showing on the left side of the graph the sporadic and mostly unipolar electrical activity. (**c**) Zoom-in to the sporadic and asynchronous regime showing a typical unipolar negative spike. (**d**) Quasi-periodic activity of PC-3 cells, presenting Biphasic spikes. (**e**) Biphasic pulses of the electrical activity of PC-3 cells, with a measurable distance between the positive and the negative pulse of about 0.3 s. (**f**–**h**) represent the characterization of the quasi-periodic activity of PC-3 cells: (**f**) Number of spikes depending on its amplitude. (**g**) Number of spikes depending on its width. (**h**) Number of spikes depending on its distance between them. The interspike intervals were distributed into time slots with a resolution of 1 s.

The electrical activity of PC-3 cells has been monitored over time. We repeated all electrical recordings at least three times for reproducibility. A baseline signal was obtained by performing electrical recordings only with medium. This experiment was made before the electrical recordings with cells took place. This results in a baseline of less than 1 pA as depicted in Figure 2a in close agreement to previous reported results [31].

PC-3 cells were then monitored in our system. Figure 2b describes the two common patterns of the current measurements at zero bias. Two typical behaviours were recorded through the experimental recordings. A basal sporadic and asynchronous activity and a quasi-periodic behaviour. A zoomed of the quasi-periodicity is presented in Figure 2c and an even larger zoom to the spike shape is given by Figure 2d. We note that the basal sporadic activities are characterized by faster and smaller magnitudes of about 100 pA, and by their asynchronous and sporadic behaviour. Yet, when quasi-periodic spikes appear, they are mostly characterized by larger signal magnitudes, slower widths ranging from 0.03 to 0.3 s and inter-spike intervals in the range of few seconds. We present a detailed statistical analysis of the recorded synchronous activity in Figure 2e–g. 

Figure 2c depicts a typical unipolar asynchronous spike observed in PC-3 cell cultures during extracellular recordings. We note that about 70% of the electrical spikes were unipolar, fast (below 200 ms) and with lower magnitudes than that of the synchronous regime. Figure 2d,e shows that quasi-periodic spikes are biphasic, and that the spike amplitude during the more intense period of electrical activity is about ±150 pA. We further analyse our data by plotting the histograms of all our synchronous signals during 3 different experiments. Figure 2f–h shows the statistics of the time between consecutive spikes, signal magnitudes and spike widths during the synchronous activity. Due to a low number of events, the histograms in Figure 2f–h shows only spikes over ±50 pA. Near half of the spikes accounted have an amplitude between ±100 and ±200 pA, and a remarkable amount of spikes exist with an even higher amplitude with maximums over 300 pA. Interestingly, we note that in most cases the quasi-periodic spikes observed in the synchronous period have larger widths than those appearing in the asynchronous regime. About 80% of the analysed spikes in the asynchronous regime had widths between 30 and 200 ms, whereas in the synchronous regime the distribution was broader and with widths larger as depicted in Figure 2g. The time between synchronous events is depicted in Figure 2h. Here we see that the distance between spikes reaches a peak around 2–3 s although the variation expands from 1 to 10 s of interval between biphasic spikes. 

The results obtained for the asynchronous analysis reveal that during the first part of the experiment, which corresponds with this asynchronous behaviour, the amount of spikes detected is much lower (around 10 times lower) than during the synchronous analysis. Comparing the synchronous and asynchronous analysis we conclude that the amount of spikes detected during the asynchronous phase is considerably lower than the amount of spikes detected during the quasi-periodic phase. The spikes are larger and wider during the quasi-periodic activity, as expected for cooperative signals [31,32]. The signal recorded using our large electrode area is the sum of all individual cell contributions. Individual cell signals cannot be resolved with sufficient spatial information. Uncorrelated cell activity appears as noise and low magnitude asynchronous spikes. Thus, we argue that the asynchronous activity regime of Figure 2b is mostly due to uncorrelated single cell activity. The origin is likely to relate with the known expression of K^+^ channels of the Kv1.3 family as shown recently in prostate cancer cell lines via patch clamp experiments [42,43,44,45].

### 3.3. Calcium Channel Inhibition

We note that the quasi-periodic nature of signals is slow and routinely occurs during prostate cancer proliferation. Additionally, the quasi-periodicity and form of the current signal resonates well with an extracellular traveling wave across the large area electrode. Once the wave reaches the sensing electrode it raises its potential relative to the counter electrode forcing a large displacement current through the double-layer capacitance, giving rise to upward and downward current spikes corresponding to the wave entrance and exiting on the recording electrode. [31,32,41]. We recorded onward and downward spike differences between 0.7 to 2 s using 1 mm^2^ electrodes. This means that we can actually record a wave speed of a few hundreds of micrometres per second, which is in fact supported by reported Ca^2+^ waves propagation speeds [46,47]. Hence, we hypothesize that Ca^2+^ channels could be involved due to their reported periodicity, slower nature and involvement to PC-3 proliferation. Hence, in order to demonstrate a possible role of Calcium channels (Ca^2^⁺) in the recorded electrical activity, we decided to block the electrical spikes using a well-known Ca^2^⁺ inhibitor, Gadolinium (Gd^3^⁺) [48].

**Figure 3:**
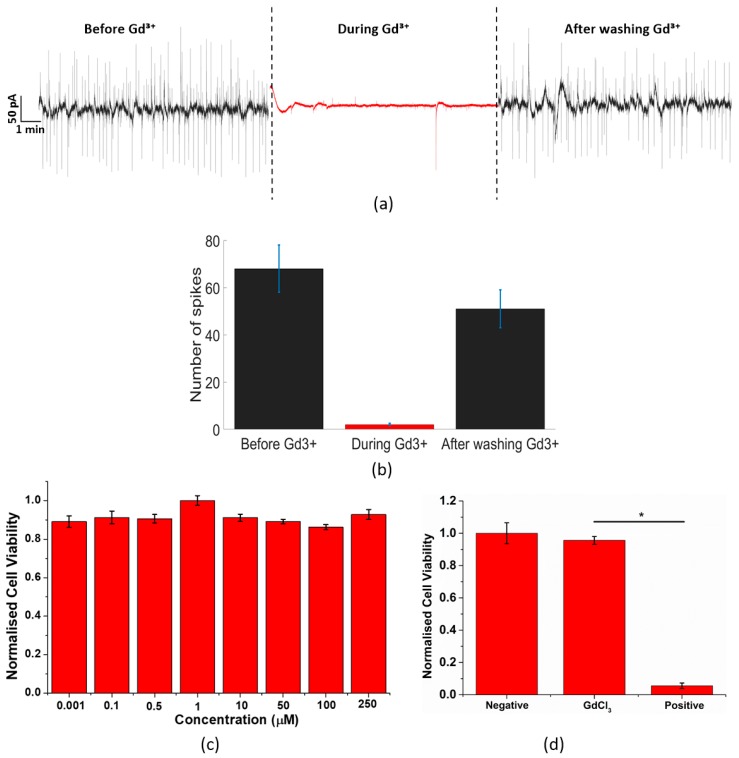
(**a**) Electrical activity of PC-3 cells culture before, during and after using Gd^3^⁺ has been used as a Ca^2^⁺ inhibitor. Before and after the use of Gd^3^⁺, the electrical activity shows quasi-periodic current oscillations. (**b**) Number of spikes detected in three different moments: before, during and after the use of the Ca^2^⁺ inhibitor. (**c**) Gadolinium chloride MTT assays. The result shows that there is no significant toxicity of GdCl^3^ in 20 min of incubation. The results of (**c**) are reported as means ±SEM. The data were analysed by one way ANOVA, *p* < 0.05, which means there is a significant difference between results of different concentrations (*n* = 3). Error bars represent standard error with respect to the repeated six measurements of the same concentration. (**d**) Positive and negative control test of gadolinium chloride. The result shows that there is no significant toxicity of 250 µM GdCl^3^ in 20 min of incubation compared with negative control (water) and positive control (250 µM triton). The results are reported as means ±SEM. (* *p* < 0.05, Student’s *t*-test). There is a significant difference between results of GdCl^3^ and positive control at 250 µM concentration (*n* = 3). Error bars represent standard error with respect to the three independent experiments.

A Ca^2^⁺ inhibitor (Gd^3^⁺) has been added during the experiment. The inhibitor has been added during 20 min and washed after that time. Gd^3^⁺ has been diluted with DI water, in concentrations between 10 and 40 µM as in previous works [40]. The medium at the beginning of the experiment was 280 µL, and we used 3 µL of Gd^3^⁺ in a concentration of 1 mM, together with the medium remained in the culture. A final concentration of Gd^3^⁺ between 10 and 20 µM was achieved. The electrical activity of PC-3 cells before adding Gd^3^⁺ is depicted in Figure 3a. Fresh PC-3 cells typically exhibiting fluctuations around 100 pA as explained in detail in Figure 2. Figure 3b demonstrates that the signal of adherent PC-3 cells could be inhibited with the addition of 20 µM of Gd^3^⁺. The inhibitor effect is fast and in less than one minute the current fluctuations lower to less than 5 pA of magnitude as given by the red trace in Figure 3a. After about 1 h the cells were washed three times with phosphate buffer saline (PBS) to remove the Gd^3^⁺ inhibitor and fresh medium was provided. After washing out, the PC-3 cells regain the original electrical activity, yielding spike magnitudes of about 100 pA as illustrated in the final black trace of Figure 3a. Additionally, gadolinium chloride MTT assays are shown in Figure 3c,d to exclude cell death after adding the inhibitor. The error reported was the standard error of the mean and shown as ±S.E.M (Figure 3c,d).

As can be seen in Figure 3, the Ca^2^⁺ channels are clearly involved in the electrical activity of PC-3 cells. Electrical activity of PC-3 cells together with Gd^3^⁺ has been recorded during about 20 min (Figure 3a red colour), reducing considerably the previous electrical activity of PC-3 cells (represented in Figure 3a in black colour in the left side of the graph). 20 min after the deposition of the inhibitor, the medium with Gd^3^⁺ was washed three times to assure the complete elimination of the inhibitor. After the washing, new medium was added and the electrical activity started firing normally (black colour in the right side of the graph) with a quasi-periodic activity. It has been demonstrated that inhibiting Ca^2^⁺ channels make the electrical activity of PC-3 cells almost disappear, confirming that these channels have a high influence in the electrical activity of this type of cells. In Figure 3b, the number of spikes detected before, during and after the use of Gd^3^⁺ are shown. As can be seen in Figure 3b, the number of spikes detected before and after the use of the inhibitor are close, in comparison with the number of spikes detected during the use of the inhibitor, which is almost zero.

In Figure 3c,d, an acute GdCl^3^ viability experiment was conducted. The results show that the cell viability of all concentrations are above 90%. Thus, it proves that GdCl^3^ will not cause the cytotoxic effect to tested cells and that the reduction of the spikes is due to the inhibition of the calcium channel instead of cell death. In Figure 3d, a positive and negative control results were compared with the GdCl^3^ results. The GdCl^3^ has a very close cell viability to the negative control, which is non-cytotoxic. While in the positive control, a cytotoxic reagent at the same concentration of the GdCl^3^ is introduced and leads to a significant reduction in the cell viability. This proves that GdCl^3^ is non-toxic to PC-3 cells during our experiments.

## 4. Conclusions

In this paper we have characterized the electrical activity of a prostate cancer cell line (PC-3) using circular gold electrodes on a silicon oxide substrate chip. PC-3 cells demonstrate a low frequency electrical activity between 0.1 Hz and 10 Hz and two distinct patterns; an asynchronous and sporadic pattern and a synchronous quasi-periodic pattern. The sporadic phase shows weak and fast spikes typically below 100 ms. Yet, the quasi-periodic phase shows spikes having an amplitude of ±150 pA, a width between 50 and 300 ms and an inter-spike interval around 0.5 Hz. The use of the well-known calcium inhibitor Gd^3^⁺ together with the slowly varying nature of the signal suggests that Ca^2^⁺ channels are involved in the electrical signalling among a population of PC-3 cells. We therefore demonstrated for the first time the feasibility of a real time and highly sensitive system to understand the signalling pathways among prostate cancer cells.

## Figures and Tables

**Table 1:** Average, S.E.M, normalized average and normalized S.E.M.

	Day 1	Day 2	Day 3	Day 4	Day 8
Average	42.0000	93.7500	156.6250	285.0000	1193.6250
S.E.M	4.5591	9.4449	16.8395	43.4133	128.5749
Normalized average	0.0352	0.0785	0.1312	0.2089	1.0000
Normalized S.E.M	2.42 × 10^−3^	5.02 × 10^−3^	8.95 × 10^−3^	2.75 × 10^−2^	6.84 × 10^−2^

## References

[1] National Cancer Institute SEER Cancer Statistics Review, 1975–2015. https://seer.cancer.gov/csr/1975_2015/.

[2] Weiner A.B., Matulewicz R.S., Eggener S.E., Schaeffer E.M. (2016). Increasing incidence of metastatic prostate cancer in the United States (2004–2013). Prostate Cancer Prostatic Dis..

[3] Buzzoni C., Auvinen A., Roobol M.J., Carlsson S., Moss S.M., Puliti D., Lujan M. (2015). Metastatic prostate cancer incidence and prostate-specific antigen testing: New insights from the European Randomized Study of Screening for Prostate Cancer. Eur. Urol..

[4] Office of National Statistic (2016). Cancer Registration Statistics, England: 2018. https://www.ons.gov.uk/peoplepopulationandcommunity/healthandsocialcare/conditionsanddiseases/bulletins/cancerregistrationstatisticsengland/final2016.

[5] Dozmorov M.G., Hurst R.E., Culkin D.J., Kropp B.P., Frank M.B., Osban J., Lin H.K. (2009). Unique patterns of molecular profiling between human prostate cancer LNCaP and PC-3 cells. Prostate.

[6] (2001). Cell L. BC Cancer Agency. 2014 Nov 21; Cell L. http://capcelllines.ca.

[7] Stone K.R., Mickey D.D., Wunderli H., Mickey G.H., Paulson D.F. (1978). Isolation of a human prostate carcinoma cell line (DU 145). Int. J. Cancer.

[8] Cunningham D., You Z. (2015). In vitro and in vivo model systems used in prostate cancer research. J. Biol. Methods.

[9] Wu X., Gong S., Roy-Burman P., Lee P., Culig Z. (2013). Current mouse and cell models in prostate cancer research. Endocr. Relat. Cancer.

[10] Rea D., Del Vecchio V., Palma G., Barbieri A., Falco M., Luciano A., de Biase D., Perdonà S., Facchini G., Arra C. (2016). Mouse models in prostate cancer translational research: From xenograft to PDX. BioMed Res. Int..

[11] Pulukuri S.M., Gondi C.S., Lakka S.S., Jutla A., Estes N., Gujrati M., Rao J.S. (2005). RNA interference-directed knockdown of urokinase plasminogen activator and urokinase plasminogen activator receptor inhibits prostate cancer cell invasion, survival, and tumorigenicity in vivo. J. Biol. Chem..

[12] Harrison I.P., Selemidis S. (2014). Understanding the biology of reactive oxygen species and their link to cancer: NADPH oxidases as novel pharmacological targets. Clin. Exp. Pharmacol. Physiol..

[13] Yang Y., Karakhanova S., Werner J., Bazhin A.V. (2013). Reactive oxygen species in cancer biology and anticancer therapy. Curr. Med. Chem..

[14] Eimon P.M., Ghannad-Rezaie M., De Rienzo G., Allalou A., Wu Y., Gao M., Roy A., Skolnick J., Yanik M.F. (2018). Brain activity patterns in high-throughput electrophysiology screen predict both drug efficacies and side effects. Nat. Commun..

[15] Salvarani N., Maguy A., De Simone S.A., Miragoli M., Jousset F., Rohr S. (2017). TGF-β1 (Transforming Growth Factor-β1) Plays a Pivotal Role in Cardiac Myofibroblast Arrhythmogenicity. Circulation.

[16] Kunzelmann K. (2005). Ion channels and cancer. J. Membr. Biol..

[17] Fraser S.P., Pardo L.A. (2008). Ion channels: Functional expression and therapeutic potential in cancer: Colloquium on Ion Channels and Cancer. EMBO Rep..

[18] Cuddapah V.A., Sontheimer H. (2011). Ion channels and tranporters in cancer. 2. Ion channels and the control of cancer cell migration. Am. J. Physiol. Cell Physiol..

[19] Prevarskaya N., Skryma R., Shuba Y. (2010). Ion channels and the hallmarks of cancer. Trends Mol. Med..

[20] Prevarskaya N., Skryma R., Bidaux G., Flourakis M., Shuba Y. (2007). Ion channels in death and differentiation of prostate cancer cells. Cell Death Differ..

[21] Arcangeli A., Becchetti A. (2015). Novel perspectives in cancer therapy: Targeting ion channels. Drug Resist. Updates.

[22] Abdul M., Hoosein N. (2002). Expression and activity of potassium ion channels in human prostate cancer. Cancer Lett..

[23] Yildirim S., Altun S., Gumushan H., Patel A., Djamgoz M.B. (2012). Voltage-gated sodium channel activity promotes prostate cancer metastasis in vivo. Cancer Lett..

[24] Zhang L., Barritt G.J. (2004). Evidence that TRPM8 is an androgen-dependent Ca^2+^ channel required for the survival of prostate cancer cells. Cancer Res..

[25] Gkika D., Flourakis M., Lemonnier L., Prevarskaya N. (2010). PSA reduces prostate cancer cell motility by stimulating TRPM8 activity and plasma membrane expression. Oncogene.

[26] Wang Y., Yue D., Li K., Liu Y.L., Ren C.S., Wang P. (2010). The role of TRPC6 in HGF-induced cell proliferation of human prostate cancer DU145 and PC-3 cells. Asian J. Androl..

[27] Holzmann C., Kilch T., Kappel S., Dörr K., Jung V., Stöckle M., Peinelt C. (2015). Differential redox regulation of Ca2+ signaling and viability in normal and malignant prostate cells. Biophys. J..

[28] Buzsáki G., Anastassiou C.A., Koch C. (2012). The origin of extracellular fields and currents—EEG, ECoG, LFP and spikes. Nat. Rev. Neurosci..

[29] Szatkowski M., Mycielska M., Knowles R., Kho A.L., Djamgoz M.B.A. (2000). Electrophysiological recordings from the rat prostate gland in vitro: Identified single-cell and transepithelial (lumen) potentials. BJU Int..

[30] Rocha P.R., Schlett P., Schneider L., Dröge M., Mailänder V., Gomes H.L., Blom P.W.M., De Leeuw D.M. (2015). Low frequency electric current noise in glioma cell populations. J. Mater. Chem. B.

[31] Rocha P.R., Schlett P., Kintzel U., Mailänder V., Vandamme L.K., Zeck G., Gomes H.L., Biscarini F., De Leeuw D.M. (2016). Electrochemical noise and impedance of Au electrode/electrolyte interfaces enabling extracellular detection of glioma cell populations. Sci. Rep..

[32] Rocha P.R., Medeiros M.C., Kintzel U., Vogt J., Araújo I.M., Mestre A.L., Biscarini F. (2016). Extracellular electrical recording of pH-triggered bursts in C6 glioma cell populations. Sci. Adv..

[33] Thomas C.A., Springer P.A., Loeb G.E., Berwald-Netter Y., Okun L.M. (1972). A miniature microelectrode array to monitor the bioelectric activity of cultured cells. Exp. Cell Res..

[34] Hoogerwerf A.C., Wise K.D. (1994). A three-dimensional microelectrode array for chronic neural recording. IEEE Trans. Biomed. Eng..

[35] Nordhausen C.T., Maynard E.M., Normann R.A. (1996). Single unit recording capabilities of a 100 microelectrode array. Brain Res..

[36] Berdondini L., Overstolz T., De Rooij N.F., Koudelka-Hep M., Wany M., Seitz P. High-density microelectrode arrays for electrophysiological activity imaging of neuronal networks. Proceedings of the ICECS 2001, 8th IEEE International Conference on Electronics, Circuits and Systems (Cat. No.01EX483).

[37] Wang K., Fishman H.A., Dai H., Harris J.S. (2006). Neural stimulation with a carbon nanotube microelectrode array. Nano Lett..

[38] Spira M.E., Hai A. (2013). Multi-electrode array technologies for neuroscience and cardiology. Nat. Nanotechnol..

[39] Obien M.E.J., Deligkaris K., Bullmann T., Bakkum D.J., Frey U. (2015). Revealing neuronal function through microelectrode array recordings. Front. Neurosci..

[40] Eversmann B., Jenkner M., Hofmann F., Paulus C., Brederlow R., Holzapfl B., Gabl R. (2003). A 128/spl times/128 CMOS biosensor array for extracellular recording of neural activity. IEEE J. Solid-State Circuits.

[41] Medeiros M.C., Mestre A., Inácio P., Asgarif S., Araújo I.M., Hubbard P.C., Biscarini F. (2016). An electrical method to measure low-frequency collective and synchronized cell activity using extracellular electrodes. Sens. Bio-Sens. Res..

[42] Teulon J., Ronco P.M., Geniteau-Legendre M., Baudouin B., Estrade S., Cassingena R., Vandewalle A. (1992). Transformation of renal tubule epithelial cells by simian virus-40 is associated with emergence of Ca^2+^-insensitive K^+^ channels and altered mitogenic sensitivity to K^+^ channel blockers. J. Cell. Physiol..

[43] Sohma Y., Harris A., Wardle C.J.C., Gray M.A., Argent B.E. (1994). Maxi K^+^ channels on human vas deferens epithelial cells. J. Membr. Biol..

[44] Logsdon N.J., Kang J., Togo J.A., Christian E.P., Aiyar J. (1997). A novel gene, hKCa4, encodes the calcium-activated potassium channel in human T lymphocytes. J. Biol. Chem..

[45] Ouadid-Ahidouch H., Van Coppenolle F., Le Bourhis X., Belhaj A., Prevarskaya N. (1999). Potassium channels in rat prostate epithelial cells. FEBS Lett..

[46] Leybaert L., Sanderson M.J. (2012). Intercellular Ca^2+^ waves: Mechanisms and function. Physiol. Rev..

[47] Kuga N., Sasaki T., Takahara Y., Matsuki N., Ikegaya Y. (2011). Large-scale calcium waves traveling through astrocytic networks in vivo. J. Neurosci..

[48] Guilak F., Zell R.A., Erickson G.R., Grande D.A., Rubin C.T., McLeod K.J., Donahue H.J. (1999). Mechanically induced calcium waves in articular chondrocytes are inhibited by gadolinium and amiloride. J. Orthop. Res..

